# Regulation of ATP binding cassette transporter A1 (ABCA1) expression: cholesterol-dependent and – independent signaling pathways with relevance to inflammatory lung disease

**DOI:** 10.1186/s12931-020-01515-9

**Published:** 2020-09-25

**Authors:** Patrick He, Ingrid C. Gelissen, Alaina J. Ammit

**Affiliations:** 1 Woolcock Emphysema Centre, Woolcock Institute of Medical Research, University of Sydney, Sydney, NSW Australia; 2 School of Life Sciences, Faculty of Science, University of Technology Sydney, Sydney, NSW Australia; 3 Sydney Pharmacy School, Faculty of Medicine and Health, University of Sydney, Sydney, NSW 2006 Australia

**Keywords:** ABCA1, COPD, Inflammation, TNF, Cholesterol, Cigarette smoke

## Abstract

The role of the ATP binding cassette transporter A1 (ABCA1) in maintaining cellular lipid homeostasis in cardiovascular disease is well established. More recently, the important beneficial role played by ABCA1 in modulating pathogenic disease mechanisms, such as inflammation, in a broad range of chronic conditions has been realised. These studies position ABCA1 as a potential therapeutic target in a diverse range of diseases where inflammation is an underlying cause. Chronic respiratory conditions such as asthma and chronic obstructive pulmonary disease (COPD) are driven by inflammation, and as such, there is now a growing recognition that we need a greater understanding of the signaling pathways responsible for regulation of ABCA1 expression in this clinical context. While the signaling pathways responsible for cholesterol-mediated ABCA1 expression have been clearly delineated through decades of studies in the atherosclerosis field, and thus far appear to be translatable to the respiratory field, less is known about the cholesterol-independent signaling pathways that can modulate ABCA1 expression in inflammatory lung disease. This review will identify the various signaling pathways and ligands that are associated with the regulation of ABCA1 expression and may be exploited in future as therapeutic targets in the setting of chronic inflammatory lung diseases.

## Background

ATP binding cassette transporter A1 (ABCA1) is a transmembrane transporter protein ubiquitously expressed in human tissue, and found in abundance in the liver and lungs (reviewed in [1]). While its primary role is to maintain lipid homeostasis by controlling the efflux of cellular cholesterol and phospholipids, ABCA1 is increasingly recognised as having anti-inflammatory functions in a diverse range of diseases where inflammation is an underlying pathogenic mechanism (reviewed in [1–4]). Thus, given the role played by ABCA1 in suppressing inflammation, understanding the signaling pathways that regulate ABCA1 expression may provide potential therapeutic strategies to maintain cholesterol homeostasis and treat conditions with excessive inflammation in the future.

### ABCA1-mediated cholesterol efflux

ABCA1 transporter plays an important role in maintaining cellular cholesterol homeostasis by participating in the reverse cholesterol transport pathway (RCT) [5]. ABCA1 expressed in peripheral cells facilitates the export of cellular cholesterol to its extracellular acceptor protein apolipoprotein-A1 (apoA-1) [6]. In macrophages, ABCA1 interacts with apoA-1 to develop nascent high density lipoprotein (HDL) before interacting with ATP binding cassette transporter G1 (ABCG1) and scavenger receptor class B type 1 to from mature HDL particles [7]. Subsequently, HDL transports the cholesterol from peripheral tissues to the liver for excretion [6].

### Altered ABCA1 expression in lung diseases

Mutations in ABCA1 can cause Tangier disease, a rare genetic disorder characterized by a substantial reduction in HDL levels [8]. Studies investigating the roles of ABCA1 in RCT have since observed similarities in the phenotype of ABCA1 deficient mice compared to the phenotype of human Tangier disease [9, 10]. The phenotype of ABCA1-knockout mice macrophages in vivo included lipid accumulation and significantly reduced HDL and apoA-1 levels [9]. Lipid accumulation as a result of reduced HDL and apoA-1 in ABCA1 and ABCG1 knockout mice macrophages was found to induce increased circulating pro-inflammatory cytokines, displaying signs of systemic inflammation [9, 10]. In addition, while similar outcomes were found in vitro with the knockdown of ABCA1 in human macrophages, ABCG1 expression was increased [11].

Evidence from both in vitro and in vivo studies have shown ABCA1 expression is altered in lung diseases such as COPD [12, 13]. In a recent study, Sonett et al. found that the expression of ABC transporters (ABCA1 and ABCG1) in lungs of patients with moderate to severe COPD to be significantly downregulated compared to healthy lungs [12]. In addition, greater downregulation of ABCA1 expression was observed compared to ABCG1 indicating that the importance of ameliorating downregulated ABCA1 may outweigh downregulated ABCG1 [12]. Cigarette smoke is proposed as the primary cause of downregulated ABCA1 expression in patients with COPD [12]. There is also evidence that cigarette smoke modulates the signaling pathways that regulate ABCA1 expression in macrophages in vitro and in vivo [12, 14]. Pulmonary abnormalities in ABCA1 expression have also been found in pneumonia caused by chlamydia pneumoniae bacteria [13, 15]. Chlamydia pneumoniae infection was demonstrated to affect cholesterol trafficking, associated with accelerating intracellular cholesterol accumulation by downregulating ABCA1 expression in mice models in vivo and human macrophage and lung epithelial cells in vitro [13, 15, 16]. However, the exact role of ABCA1 in the pathogenesis of respiratory infection is currently unknown.

### ABCA1, cholesterol homeostasis and the link with inflammation

Cellular cholesterol is linked with numerous inflammatory functions of the lungs [17]. Hence, the proteins involved in the process of RCT may impact the inflammatory responses elicited by overloading of free cholesterol in macrophages [18]. Understanding the functions of ABCA1 and HDL may reveal the impact of regulating cholesterol on inflammatory functions in lungs. In addition to HDL’s role in RCT, HDL assists with the production of surfactant in alveolar type II cells [19], a critical cell type in COPD. Furthermore, it is known that when ABCA1 is repressed, excess cholesterol builds up in alveolar cells, damaging surfactant function and increasing the inflammatory response which has been implicated in the pathogenesis of chronic obstructive pulmonary disease (COPD), asthma and other lung diseases (reviewed in [1, 17, 20]).

Considering the relationship between cholesterol homeostasis and airway inflammation, the therapeutic focus has been on reducing intracellular cholesterol synthesis using cholesterol-lowering drugs, like statins [17]. There have been large clinical trials, including STATCOPE, that have tested the effectiveness of statin treatment on patients with COPD [21, 22]. Despite the benefits that were observed in prior retrospective studies, STATCOPE observed no therapeutic benefit from statin treatment in patients with COPD [21, 23, 24]. This would suggest that a different approach to restoring cholesterol balance, perhaps through alternative targets that have the potential to modulate cholesterol, and repress inflammation at the same time, such as ABCA1, warrant further investigation. This is the focus of this review.

Most studies exploring the role and function of ABCA1 have been conducted in cholesterol associated disease states such as atherosclerosis [25]. Atherosclerosis was traditionally known as a cholesterol storage disease [26]. However, it is now recognised as a chronic inflammatory disease, with evidence of excess cholesterol accumulation promoting inflammatory responses [27, 28]. There are a number of lines of evidence from clinical and preclinical studies that link atherosclerosis and inflammation. An earlier study using mice models showed that inhibiting the expression of inflammatory mediators significantly improves the severity of atherosclerosis [29]. Patients with atherosclerosis are identified by high levels of low density lipoproteins (LDL) cholesterol in their body [30]. Regulation of LDL cholesterol homeostasis affects the innate immune system [31]. This is shown by LDL cholesterol inducing pro-inflammatory cytokines through activation of the toll-like receptor (TLR) pathways [32, 33]. This has led the research focus to shift towards looking at the anti-inflammatory benefits of ABCA1 in atherosclerosis. ABCA1 may also exert direct anti-inflammatory actions [34, 35] that are independent of its impact on cholesterol homeostasis, although given the central role of cholesterol in cell survival, this independence may prove difficult to tease out.

Nevertheless, growing evidence has clearly implicated ABCA1 as a target of interest in diseases typified by inflammation. The potential anti-inflammatory effects of ABCA1 have thus led to increasing interest towards investigating the benefit of ABCA1 in inflammatory lung diseases [12, 36]. While it is well recognised that cholesterol exerts significant regulatory control over ABCA1 expression, less well understood is the impact that inflammatory mediators themselves have on ABCA1 upregulation. Given the interaction between cholesterol homeostasis and pulmonary inflammation, the linkage represents a feasible option for therapeutic intervention with the potential to have beneficial effects in both atherosclerosis and COPD; two chronic conditions that are a common comorbidity. To highlight these potential therapeutic interactions, the aim of this review is to explore the molecular mechanisms responsible for ABCA1 expression. Notably, ABCA1 expression can be regulated via cholesterol-dependent and -independent mechanisms and the aim of the review is to explore these signaling pathways in the context of respiratory disease.

### Regulation of ABCA1 expression: cholesterol-dependent signaling pathways

**Fig. 1:**
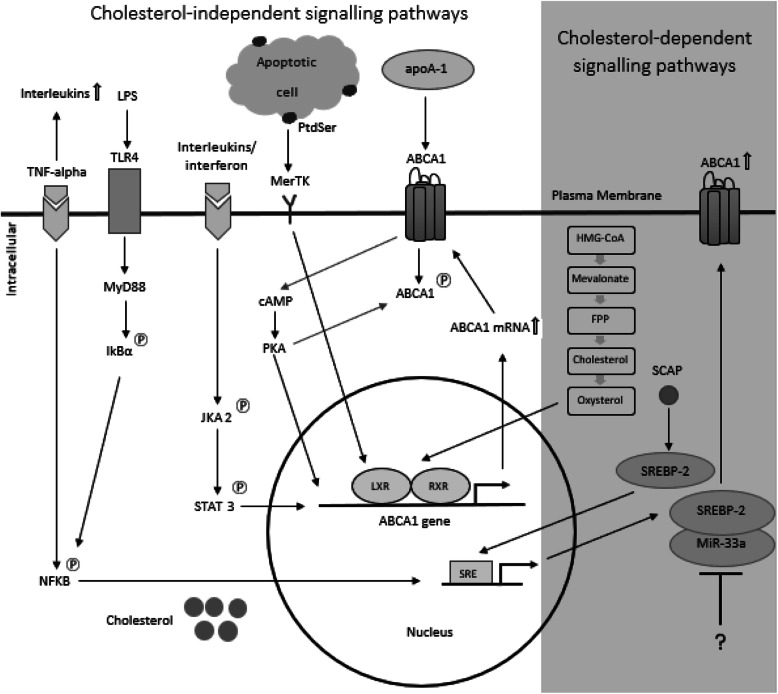
ABCA1 cell signaling pathways. The cholesterol-dependent signaling pathways which regulate ABCA1 include the LXR/RXR and SREBP-2 pathway. These two pathways are activated in response to changes in intracellular cholesterol levels by oxysterols and SREBP-2 molecules which target the transcriptions factors, LXR/RXR and SREBP-2, respectively. The cholesterol-independent signaling pathways associated with ABCA1 regulation include the NF-κB, TLR4/Myleoid88, JAK2/STAT3, cAMP/PKA, apoptosis pathways involved in regulating inflammatory responses

Cholesterol homeostasis is maintained through feed-forward and feed-back mechanisms involving ABCA1 and other partners. Notably, ABCA1 expression is regulated through key signaling pathways mediated by excess cholesterol (Fig. 1), which will be described briefly below.

#### LXR/RXR pathway

Cholesterol in cells can be converted into oxysterols, which in turn can potently upregulate ABCA1 expression [4]. In cell types with relevance to inflammatory lung diseases (including macrophage, epithelial and smooth muscle cells (Table 1) [4, 35, 40–43]), the predominantly reported oxysterol is 25-hydroxycholesterol (25-HC). This naturally occurring stimulus increases the expression of ABCA1 through activation of liver X receptor (LXR) pathway [44, 45]. LXR forms a heterodimer with retinoid X receptor (RXR), and together they form a transcription factor that binds to the promoter element in the ABCA1 gene to increase expression and ultimately the upregulation of ABCA1 protein expression [44, 45], amongst other targets. Transcription occurs upon influx of excess intracellular cholesterol, however cholesterol does not directly activate this pathway but is converted to 25-HC [46]. While oxysterols are the ligands that activate LXR/RXR to increase ABCA1 expression, cholesterol loading triggers the increase in oxysterol production downstream of the mevalonate pathway to initiate increasing transcription of ABCA1 gene [47, 48]. Specific oxysterols can be produced de novo in the mevalonate pathway, independent of the common cholesterol derived oxysterol [49]. Cholesterol in humans is predominately synthesized de novo by the liver, initiated by acetyl coenzyme 3-hydroxy-3-methyl-glutaryl-coenzyme [50]. 24(*S*),25-epoxycholesterol (24,25EC) is such an example, where it is produced from acetyl coenzyme during a gap in the mevalonate pathway [51]. Similar to other oxysterols, 24,25EC has since shown in studies to activate LXR receptors [52].

**Table 1:** Currently known effects of clinical and experimental drugs on ABCA1 expression in cells in vitro

Cell Type	Stimuli	Change in ABCA1 Protein Expression	References
Macrophage Cells	Simvastatin Atorvastatin TNF Cholesterol/CD Oxysterol (25 Hydroxyl Cholesterol) Cigarette Smoke Extract	↓ ↓ ↓/↑ ↑ ↑ ↓	[37, 38] [37, 38] [16, 39] [40] [41] [12]
Bronchial Epithelial Cells	Simvastatin Atorvastatin TNF Cholesterol/CD Oxysterol (25 Hydroxyl Cholesterol)	ns ns ↑ ↑ ↑	[4] [4] [4] [4] [4]
Airway Smooth Muscle Cells	Cholesterol/CD	↑	[42]

Given the important role played by LXR in this pathway, it follows that a potential therapeutic target to modulate cholesterol homeostasis is by increasing ABCA1 expression through the LXR dependent pathway [41]. To this end, a series of synthetic LXR agonists have been developed that are able to significantly increase ABCA1 expression [42]. However LXR agonist treatment outcomes have been varied with reports of adverse off-target effects, including increased fatty acid, triglyceride synthesis and hepatic steatosis [53–55]. Recently, there has been increasing interest in the possibility that LXRα, an isoform of LXR, is the primary cause for adverse effects of conventional LXR ligands [56]. This has created interest in the development of LXRβ-specific drugs that are thought not to generate such adverse effects.

#### SREBP-2 pathway

Another pathway involved in the regulation of cellular cholesterol homeostasis is mediated by the sterol regulatory element binding protein 2 (SREBP-2) transcription factor [44]. SREBP-2 regulates ABCA1 independently of the LXR pathway, and, unlike the LXR pathway activation, SREBP-2 activation can decrease ABCA1 expression [46, 56, 57]. SREBP-2 activation is dependent on the SREBP cleavage activating protein (SCAP) protein, which has a sterol sensing domain capable of sensing cellular cholesterol levels [46]. When intracellular cholesterol levels are low, SCAP escorts SREBP-2 to the Golgi for activation before being translocated to the sterol regulatory element (SRE) in the nucleus to promote transcription of different target genes as well as SREBP-2 itself [33]. Located within intron 16 of SREBP-2 is microRNA-33a (miR-33a), which is co-transcribed with SREBP-2 [33, 58]. The miR-33a has been shown to inhibit ABCA1 expression in mice pancreatic islets ex vivo and human macrophages in vitro [58–60]. Recently, it has been demonstrated that inhibition of miR-33a in mice hepatic tissue and macrophages in vivo leads to an increased expression of the miR-33a target gene ABCA1 [61, 62]. Increasing ABCA1 expression by inhibiting miR-33a allows ABCA1 target treatment to bypass the adverse effects of current treatments that activate the LXR pathway [62].

### Regulation of ABCA1 expression: cholesterol-independent signaling pathways

Recent investigations have revealed that a number of inflammatory mediators and noxious exposures (such as cigarette smoke) that drive lung disease also have an impact on ABCA1 expression. Clinically-used and experimental drugs also target ABCA1, with some key in vitro studies performed in cells with relevance to respiratory disease listed in Table 1. These changes to ABCA1 expression are regulated by signaling (Fig. 1) (as detailed below), some of which are subject to cross-talk.

#### TNF

Tumour Necrosis Factor (TNF) is a proinflammatory cytokine that is highly expressed in patients with COPD [63]. TNF is known to activate many signaling pathways of which some have previously shown to induce changes in ABCA1 expression [64]. These pathways include the nuclear factor-κB (NF-κB), sterol regulatory element binding protein 2 (SREBP-2) and janus kinase 2/signal transducer 3 (JAK2/STAT3) [65–67]. With numerous pathways and molecular partners involved, studies have shown that TNF stimulation can result in different impacts on ABCA1 expression depending on the cell line [4, 16, 39, 68]. For example, TNF stimulation can increase ABCA1 expression via the canonical NF-κB pathway [69]. However, activation of NF-κB induced by TNF also upregulates the mRNA levels of miR-33a by targeting its host gene SREBP-2 in macrophages in vitro and mice in vivo [58]. As increased expression of miR-33a is associated with repressed ABCA1 expression, increasing TNF in this context will result in decreased ABCA1 expression [70]. We have recently shown that TNF increases ABCA1 protein upregulation in BEAS-2B epithelial cells in vitro [4], however to date, the molecular mechanisms for these findings are unknown. Interestingly, studies performed in parallel in another lung epithelial cell line, A549 in vitro, indicated that TNF did not increase ABCA1 protein in these cells [4]. Perhaps the difference in impact of TNF on ABCA1 within the two lung epithelial cell lines, used commonly as cellular models of respiratory inflammation, are the different contribution of miR-33a or other known ABCA1 regulators. Further studies are warranted to establish the mechanisms involved.

#### TLR4/Myeloid88 pathway

Toll like receptor 4 (TLR4) is a primary source of innate immune system defence [71]. This protein is expressed in macrophages and lung bronchial epithelial cells, two key cell types driving airway inflammation [72]. The major cause of airway inflammation in patients with COPD is cigarette smoke [72]. Notably, cigarette smoke exposure (CSE) results in an increase in lipopolysaccharide (LPS)-induced TLR4 expression shown in primary small airway epithelial cells in vitro and mice in vivo [72, 73]. The LPS dependent increase in TLR4 activates its downstream adaptor molecule Myeloid88 that transduces TLR4 signaling to the NF-κB inhibitor, IκBα [74]. Phosphorylation of IκBα allows NF-κB to translocate into the nucleus [75], ultimately activating inflammatory genes responsible for encoding proinflammatory cytokines like TNF, while also promoting the expression of miR33a which reduces ABCA1 expression [69, 76]. The effect of cigarette smoke on the TLR4/Myeloid88 pathway was explored by exposing human macrophage THP-1 cells in vitro and mice macrophages in vivo to CSE [10, 12, 14]. The results showed that ABCA1 expression was significantly downregulated by CSE exposure in vitro and in vivo [10, 12, 14].

#### cAMP/PKA pathway

The cyclic adenosine monophosphate (cAMP)/protein kinase A (PKA) pathway is responsible for regulating multiple cellular functions, including anti-inflammatory responses [53]. Activation of cAMP/PKA pathway reduces inflammation by inhibiting NF-κB, preventing the reduction of ABCA1 and increased pro-inflammatory cytokines caused by NF-κB [69, 77]. ABCA1 expression is also regulated both transcriptionally and post-translationally by the cAMP/PKA pathway [55]. Transcriptionally, cAMP induces an increase in ABCA1 expression and its mediated reverse cholesterol transport [55, 78]. Prior studies have shown that stimulating human fibroblasts in vitro and murine macrophages in vivo with a 8-bromo-cAMP analog activated the cAMP/PKA pathway [8, 79]. Early studies performed on human macrophages were, however, unable to show a significant increase in ABCA1 expression via activation of the cAMP/PKA pathway [40, 80, 81]. In contrast, a recent study performed by Liao et al. showed that stimulating human THP-1 macrophage cells with intermedin, a calcitonin family peptide, significantly increased ABCA1 expression and cholesterol efflux through the cAMP/PKA pathway [23]. It is important to note that the impact of cAMP on ABCA1 expression depends on the species used for the study, as there are key differences in the 5′-promoter element of the murine and human ABCA1 gene whereby the functional cAMP response element (CRE) in murine ABCA1 gene promoter is not present in the first intron of the human promoter [82, 83].

However, while transcriptional regulation differs, cells from both human and mice are responsive to cAMP, albeit via dissimilar mechanisms. Post-translationally, the upregulation of ABCA1 phosphorylation and cholesterol efflux in this signaling cascade is initiated when apoA-1 interacts with ABCA1 during the process of reverse cholesterol transport [78]. The interaction between apoA-1 and ABCA1 activates heterotrimeric G proteins and adenylyl cyclase to produce cAMP, consequently activating PKA, a direct downstream target of cAMP [43, 78, 84]. PKA then increases phosphorylation of ABCA1, which has been shown by previous studies to significantly increase ABCA1 expression and cholesterol efflux in macrophage cells [78, 85]. Whether upregulation of ABCA1 by the cAMP/PKA pathway occurs in the context of inflammatory lung diseases warrants further investigation, especially since cAMP-elevating agents (e.g. β_2_-adrenergic receptor agonists and phosphodiesterase inhibitors) are widely used treatments in respiratory medicine today.

#### JAK2/STAT3 pathway

Signal transducer and activator of transcription 3 (STAT3) plays a complex role in regulating inflammation that occurs with airway injury [86]. Cigarette smoke is associated with the occurrence of airway injury and inflammation, and can also activate STAT3 in the lungs [86, 87]. While the exact role of STAT3 in inflammatory lung diseases is relatively poorly understood, it is known that the absence of STAT3 expression impairs the innate anti-inflammatory response of the lungs [86], suggesting that the presence of STAT3 is important in reducing lung inflammation. Thus far, evidence has shown that STAT3 activation as a result of ABCA1 and apoA-1 interaction produces an anti-inflammatory effect [88]. Similar anti-inflammatory effects were shown by pro-inflammatory cytokines, interleukins and interferons activation of the janus kinase 2 (JAK2)/STAT3 pathway [89, 90]. A known cytokine that activates this pathway is interleukin 6 (IL-6), with IL-6 expression promoted by inflammatory markers of COPD like TNF [89, 91]. The initial signaling molecule which is activated upon apoA-1 and ABCA1 interaction or by IL-6 cytokine is JAK2 [88, 92]. Upon activation of JAK2, it undergoes autophosphorylation, before phosphorylating its downstream target STAT3 [92, 93]. In human and mouse macrophages, phosphorylated STAT3 binds onto the CRE site located on the first intron of the human and murine ABCA1 gene to increase the gene expression of ABCA1 [90, 94].

#### Apoptosis increases ABCA1 expression

During acute and chronic airway inflammation there is a significant increase in immune and structural cells that undergo apoptosis [95]. The clearance of cell apoptosis in the lungs is primarily regulated by airway macrophages in a process called efferocytosis [95]. Apoptotic cells have previously been shown to be directly associated with an increase in ABCA1 expression in macrophages [96, 97]. Apoptotic cells contain phosphatidylserine (PtdSer) that acts like a recognition signal for phagocytes, binding onto cell surface receptors on macrophages, like mer tyrosine kinase (MerTK) [95, 97]. During efferocytosis regulated by MerTK in pulmonary tissue, the LXR pathway is activated; thus expression of ABCA1 is upregulated [90]. With the increased apoptosis that occurs in inflammatory lung diseases like COPD, the likely corollary is that ABCA1 expression should be increased; however, this prediction does not hold true because the process of efferocytosis in patients with COPD is dysregulated [98]. The exact mechanism underlying the defect of efferocytosis in COPD is unknown, however, it is speculated to be linked to alterations in expression and function of the PtdSer receptors such as MerTK [95, 99]. Hence, additional studies exploring the molecular mechanisms behind dysregulated efferocytosis in COPD are needed to identify what the potential effect of functional alterations in MerTK has on ABCA1 expression.

Recent studies have reported a pathway regulated by the phagocytic receptor brain specific angiogenesis inhibitor 1 (BAI1), operates in a similar manner to the way in which MerTK recognises the PtdSer on apoptotic cells [100]. In brief, BAI1 activates its downstream signaling mediator Ras-related C3 botulinum toxin substrate 1 (RAC1) upon contact with PtdSer to promote ABCA1 transcription independent of the LXR pathway [100]. It is currently unclear whether this pathway is present in pulmonary tissue. Thus, further studies examining the effect of apoptosis on ABCA1 expression in lung macrophages are warranted.

#### ApoA-1 interaction with ABCA1

ApoA-1 is an acceptor for cellular cholesterol from peripheral cells that directly interacts with ABCA1 to forms nascent lipid-poor HDL [101]. Murphy et al. showed that HDL formed from the interaction between apoA-1 and ABCA1 had anti-inflammatory effects [102]. It is likely that the anti-inflammatory properties of apoA-1 are directly associated with regulation of the ABCA1 signaling pathways, cAMP/PKA and JAK2/STAT3 [78, 88]. As outlined above, both of these signaling pathways are known to upregulate ABCA1 expression, with increased ABCA1 expression correlated with increase in HDL [20]. The signaling pathways involved with the regulation of ABCA1 expression are hence key in mediating the anti-inflammatory properties of HDL.

Intriguingly, apoA-1 represses LPS-induced proinflammatory cytokine production via the TLR4/myeloid88 pathway in macrophages [34]. This was linked to reduction in mRNA stability of proinflammatory mediators through the actions of the destabilising RNA binding protein, tristetraprolin (TTP) [34]. While it is unclear as to the exact mechanism by which the interaction between ABCA1 and apoA-1 induce the TTP-mediated anti-inflammatory effect, Yin et al. reported that the effect of apoA-1 on TTP was significantly diminished when ABCA1 expression was silenced [34]. Taken together, these studies underscore the promise of ApoA-1 as a beneficial target in respiratory disease. However, the cost of purified apoA-1 and recombinant apoA-1 may prove prohibitive to further investigation. Synthetic analogs of apoA-1, known as apoA-1 mimetics, have been developed that offer a cost-effective solution [103]. Excitingly, ApoA-1 mimetics 5A and 4F administered intravenously and intratracheally have been shown to exhibit anti-inflammatory and antioxidant effects in human coronary artery cells in vitro and mice in vivo through interaction with ABCA1 [103, 104]. Further investigations are warranted.

### Effect of current medications in pulmonary diseases on ABCA1 expression

Current pharmacological treatments for people with respiratory disease include the use of corticosteroids, bronchodilators (such as β_2_-adrenergic receptor agonist) and phosphodiesterase inhibitors. Although some studies have examined the impact of current respiratory medications on ABCA1 expression, there is much scope for further investigation. The effect of these medicines on the signaling pathways regulating ABCA1 expression will be outlined below.

#### Corticosteroids

To date, there have been limited studies aimed at examining the effect of steroids on ABCA1 expression. While there is no evidence of a glucocorticoid response element in the ABCA1 promoter, non-*cis* mediated transcriptional regulation of ABCA1 has been shown to exist in macrophages [105, 106]. The steroid dexamethasone decreased ABCA1 expression through a LXR-independent pathway in macrophages in vitro [106].

#### Bronchodilators

β_2_-adrenergic receptor agonists are commonly used bronchodilators for treating lung diseases such as COPD or asthma [107]. The mechanism by which β_2_-agonists promote bronchodilator effects in the lungs is thought to occur via cAMP/PKA-dependent reduction in intracellular calcium [108], although other pathways may exist. Activation of the cAMP/PKA pathway is the result of increased intracellular cAMP after β_2_-agonists treatment [109]. As outlined in a previous section, it has been established that cAMP induces upregulation of ABCA1 expression in human macrophages [110]. Thus, in addition to the bronchodilator effects of β_2_-agonists treatment, anti-inflammatory properties of β_2_-agonists may be associated with ABCA1 mediated suppression of inflammatory responses in the lungs.

#### Phosphodiesterase inhibitors

Type 4 phosphodiesterase (PDE4) inhibitors are a treatment option in COPD. Similar to the effect of β_2_-agonists, PDE4 inhibitors exhibit multiple benefits which include reducing inflammation and eliciting airway smooth muscle relaxation via the cAMP/PKA pathway [18]. The PDE4 selective inhibitor, rolipram, has been shown to increase intracellular cAMP which upregulates ABCA1 expression and apoA-1-mediated cholesterol efflux in mouse and human macrophages [111].

### ABCA1 as an alternative biological target for the treatment of lung inflammation

#### Statins

Statins were postulated as possible treatment for inflammatory lung diseases including COPD. However, clinical trials like STATCOPE were shown to have an overall insignificant impact on patient outcomes [21, 112, 113]. Previous studies have looked at whether the reason behind the statins’ inability to significantly reduce inflammation during pulmonary exacerbation is linked with ABCA1 being downregulated as a result of statin treatment [1, 4, 37]. We recently showed that in human lung epithelial cell lines in vitro, both simvastatin and atorvastatin did not significantly impact on ABCA1 expression (Table 1) [4]. In human macrophage cells in vitro*, *however, Niesor et al. showed that simvastatin and atorvastatin reduced ABCA1 expression and increased miR33, supporting the possibility that a repressive effect on ABCA1-mediated anti-inflammatory functions could have played a role in the equivocal outcomes shown in STATCOPE [37]. It is plausible that the different results in these cell lines were due to cell type specificity. Hence, by testing this hypothesis on other pulmonary cell lines, like airway smooth muscle cells, we would improve our understanding of the effect of statins on ABCA1 expression more broadly, and whether combination therapy to ameliorate possible statin-induced ABCA1 downregulation is suitable.

#### LXR agonists

Given that the LXR transcription factor is a potent inducer of ABCA1 expression coupled with evidence of anti-inflammatory impact [11], it follows that LXR agonists would be strong candidates as potential therapeutics for treatment of inflammatory lung disease. This was supported by recent studies using intranasal administration of LXR agonists (T0901317 or GW3965) that showed significant attenuation of pro-inflammatory cytokines in mice in vivo and lung epithelial cells in vitro [114, 115]. However, as outlined, currently available LXR ligands target both LXR isoforms, leading to undesirable side effects on lipid parameters [116, 117]. These include increased plasma fatty acid and triglycerides, which are known risk factors of COPD [118–120]. Early LXR agonists, such as GW3965, have also failed to completely repress inflammatory cytokine production from alveolar macrophages in vitro [121]. The adverse effects of synthetic LXR agonist treatment, including elevated triglyceride levels and hepatoxicity, are thought due to LXRα activation [117]. Thus, development of LXRβ-selective agonists are currently under clinical investigation to mitigate against hepatic lipogenesis.

## Conclusions

In recent years, our understanding of the role and function of ABCA1 has evolved from the traditional function in maintaining cellular cholesterol homeostasis to include its potent anti-inflammatory function. This has led to the possibility of exploiting known and novel signaling pathways to upregulate ABCA1 expression in a number of chronic disease settings. Notably, signaling pathways implicated in inflammatory responses are responsible for regulating ABCA1 (transcriptionally or post-translationally) and its ability to repress inflammation exists. Currently unknown is the extent of the therapeutic benefit that may be achieved by targeting ABCA1 in inflammatory lung diseases like COPD. Despite the primary focus on the potential of ABCA1 as a therapeutic target in atherosclerosis, its dual role in repressing inflammation while maintaining cholesterol homeostasis represents a promising therapeutic target for inflammatory lung diseases in the future.

## Data Availability

Not applicable.

## Abbreviations

ABCA1: ATP binding cassette A1
apoA-1: Apolipoprotein A1
BAI1: Brain specific angiogenesis inhibitor 1
cAMP: Cyclic adenosine monophosphate
COPD: Chronic obstructive pulmonary disease
CRE: Camp response element
CSE: Cigarette smoke extract
25-HC: 25-hydroxycholesterol
HDL: High density lipoprotein
IL: Interleukin
JAK2: Janus kinase 2
LPS: Lipopolysaccharide
LXR: Liver X receptor
MerTK: Mer tyrosine kinase
miR-33: Micro RNA-33
NF-κB: Nuclear factor κB
PKA: Protein kinase A
PtdSer: Phosphatidylserine
RAC1: Ras-related C3 botulinum toxin substrate 1
RCT: Reverse cholesterol transport
RXR: Retinoid X receptor
SCAP: SREBP cleavage activating protein
SRE: Sterol regulatory element
SREBP-2: Sterol regulatory element binding protein 2
STAT3: Signal transducer 3
TLR4: Toll like receptor 4
TNF: Tumour necrosis factor
TTP: Tristetraprolin
